# Fast to Forgive, Slow to Retaliate: Intuitive Responses in the Ultimatum Game Depend on the Degree of Unfairness

**DOI:** 10.1371/journal.pone.0096344

**Published:** 2014-05-12

**Authors:** Eamonn Ferguson, John Maltby, Peter A. Bibby, Claire Lawrence

**Affiliations:** 1 Personality, Social Psychology and Health (PSPH) group, School of Psychology, University of Nottingham, Nottingham, United Kingdom; 2 School of Psychology, University of Leicester, Leicester, United Kingdom; University of Goettingen, Germany

## Abstract

Evolutionary accounts have difficulty explaining why people cooperate with anonymous strangers they will never meet. Recently models, focusing on emotional processing, have been proposed as a potential explanation, with attention focusing on a dual systems approach based on system 1 (fast, intuitive, automatic, effortless, and emotional) and system 2 (slow, reflective, effortful, proactive and unemotional). Evidence shows that when cooperation is salient, people are fast (system 1) to cooperate, but with longer delays (system 2) they show greed. This is interpreted within the framework of the social heuristic hypothesis (SHH), whereby people overgeneralize potentially advantageous intuitively learnt and internalization social norms to ‘atypical’ situations. We extend this to explore intuitive reactions to unfairness by integrating the SHH with the ‘fast to forgive, slow to anger’ (FFSA) heuristic. This suggests that it is advantageous to be prosocial when facing uncertainty. We propose that whether or not someone intuitively shows prosociality (cooperation) or retaliation is moderated by the degree (certainty) of unfairness. People should intuitively cooperate when facing mild levels of unfairness (fast to forgive) but when given longer to decide about another's mild level of unfairness should retaliate (slow to anger). However, when facing severe levels of unfairness, the intuitive response is always retaliation. We test this using a series of one-shot ultimatum games and manipulate level of offer unfairness (50:50 60:40, 70:30, 80:20, 90:10) and enforced time delays prior to responding (1s, 2s, 8s, 15s). We also measure decision times to make responses after the time delays. The results show that when facing mildly unfair offers (60:40) people are fast (intuitive) to cooperate but with longer delays reject these mildly unfair offers: ‘fast to forgive, and slow to retaliate’. However, for severely unfair offers (90:10) the intuitive and fast response is to always reject.

## Introduction

The explanation for widespread human cooperation is a challenge to science. While a number of mechanisms (e.g., reciprocity, kin selection) have been suggested to explain cooperation [1], these do not explain cooperation between anonymous strangers where the possibility for reciprocity is absent, such as in lab based one-shot economic games or blood donation [2]–[3].To account for cooperation in these contexts researchers have focused on the role of intuitive and emotional processes [2], [4]. One line of research has extended the dual systems framework of system 1 (fast, intuitive, automatic, effortless, emotional) and system 2 (slow, reflective, effortful, proactive and unemotional: [5]) to cooperation [6]. These two systems can sometimes be in conflict (suggesting different actions) or sometimes congruent (predicting the same action) [5]. Indeed, Rand et al [6]–[7] showed that systems 1 and 2 were in conflict with respect to prosocial behavior. They showed that people were more cooperative in anonymous one shot public goods game (PGG) when making decisions quickly, but exhibited greed with longer delays. To explain this finding, these authors propose the ‘social heuristics hypothesis’ (SHH) [2], [6]. The SHH is based on learning, internalization and automization of social norms. People intuitively learn what is potentially an advantageous strategy in everyday life and over-generalize this to ‘atypical’ situations [2], [7]. For example, it is advantageous to cooperate in everyday situations where others are also likely to reciprocate. Thus cooperation becomes the fast intuitive response that is over generalized to an atypical context that emphasizes cooperation (e.g., a one-shot PGG) but where reciprocity is not possible. However, when one expects cooperation, but is treated unfairly, then retaliation is the expected intuitive response [2]. The SSH also proposes that the expression of the intuitive response is modifiable by experience [2], [6]–[7]. We propose that the degree of unfairness is one such modifying factor [8] and that people are more likely to be intuitively prosocial when facing mild levels of unfairness and to intuitively retaliate when facing severe unfairness.

### Time, Cooperation and Punishment: Degrees of Fairness

Within the framework of the SHH Rand et al [2], [6] argue it is advantageous to retaliate or defect when facing unfairness, as this instills norms of fairness and ensures higher offers in the future. Thus levels of retaliation for unfair offers should be higher when decisions are made quickly. Consistent with this are findings from neuroeconomics that show that intuitive processes underlie the rejection of unfair offers in an ultimatum game (UG) [9]. Furthermore, behavioral data from Rand and Nowak [2] showed, using a repeated Prisoner's Dilemma Game, that if a partner was transgressed against (their partner defected) on the previous round, faster responses on the subsequent round were associated with reduced cooperation, but if they were cooperated with on the previous round faster responses were associated with greater cooperation [2]. Thus when facing a transgression to intuitive response is to retaliate and when facing cooperation it is to cooperate.

However, the SHH suggests that intuitive responses are modifiable by experience [2], [7]. As such, in the experiment reported here we extend this key finding by exploring a crucial experiential factor that should influence whether someone is intuitively cooperative or retaliatory when facing unfairness: the degree of unfairness [8]. When a transgression is mildly unfair, evolutionary accounts suggest that behaving cooperatively may potentially stop the situation escalating by being reparative as well as enhance the responder's good reputation [10]–[12]. Thus, when faced with mild levels of unfairness the intuitive and fast response should be to act prosocially and cooperate. However, for severe levels of unfairness the fast response should be retaliatory.

We test this hypothesis using the ultimatum game (UG) which allows us to examine how people respond to varying levels of unfairness in a series of anonymous one-shot interactions. In the UG a proposer decides how much to share with the responder. The responder has the option to accept or reject this offer. If they reject the offer, both parties get nothing, if they accept both parties receive the proposed amounts. The rational (system 2) economic model suggests that people should accept all offers, as having even 10% of an endowment is better than nothing. However, people respond to offers of increasing unfairness with higher rejection rates [13]. Indeed, a 60:40 offer (60% to the proposer and 40% to the recipient) is clearly less unfair than a 90:10 offer (90% to the proposer and 10% to the recipient) (see Figure S1 for supporting evidence).

Mild transgressions (e.g., 60:40 offers) may also represent a degree of uncertainty about the proposer's intentions, as well as how the responder should act. Considering such uncertainty Fudenberg, Rand and Dreber [14] state that ‘… in an uncertain world it can be payoff-maximizing to be slow to anger and fast to forgive’ (p 742: italics added). Such a ‘fast to forgive, slow to anger’ heuristic would suggest that, for mildly unfair offers, when people respond quickly acceptance rates should be higher (‘fast to forgive’) but given longer to decide acceptance rates should be lower (‘slow to anger/retaliate’). However, when the offer is clearly unfair (90:10) acceptance will never be an intuitive option [2] and people will always reject all offers regardless of time delays. We term this combination the SHH-fast to forgive slow to anger (or SHH-FFSA) hypothesis.

In contrast to this SHH-FFSA hypothesis, two alternative hypotheses can be suggested based on the ‘reflective model’ of prosociality [4]. The reflective model suggests that individuals must overcome impulses (which may be selfish) in order to act prosocially and system 2 offers the mechanism to control these impulses. It has been argued that a selfish impulse in the UG reflects a preference to maximize personal gain by accepting unfair offers, but that this is in conflict with culturally dependent norms of fairness, and is controlled by system 2 [15]. That is, system 2 overrides the selfish impulses in order to reject unfair offers. This account which we term the ‘*reflective economic temptation hypothesis*’, suggests that acceptance rates should decease when responders have longer to consider their options. This account has some empirical support in the neuroeconomics literature showing that the disruption of areas involved in executive control (the right dorso-lateral prefrontal cortex) result in higher acceptance rates of unfair offers [15].

An alternative view based on the reflective model is that negative emotions associated with unfairness (e.g., anger) lead to the impulse to reject (punish) unfairness [16]. That is, the fast intuitive response is to reject (retaliate) but that the rational system 2 overrides this. We term this the ‘*reflective rationality hypothesis*’ which suggest that with increased time delays acceptance rates should increase.

We test these predictions using a series of one-shot UG games, where unlike the Rand and Nowak [2] study, there is no opportunity for reciprocation. Thus, this is a strong test of the SSH as it examines if the intuitive response is observed when there is no opportunity for reciprocation or interaction. We further extend the Rand and Nowak [2] study by not only experimentally manipulating the time a person has to wait (the time delay) before being able to make a prosocial (accept) or retaliatory (reject) response but also measure how long they take, after the time delay, to make their decision (the decision time). Thus, we explore the effects of time on prosocial and retaliatory responses, to varying degrees of unfairness, by manipulating enforced time delays before respondent can accept or reject an offer and measuring individual variation in post-delay decision times to make the accept or reject decision.

### Previous Literature on Time Delays on Acceptance Rate in the Ultimatum Game

A total of 8 studies have examined the effects of time constraints in UG acceptance rates [17]–[24]. Oechssler et al [20] is an update of [19] and a study by Rubinstein [25] measured response times rather than manipulated them. The pattern of results across these studies is mixed, with some finding greater acceptance with longer delays and some no effects. This may reflect wide variation in sample sizes, task instructions, as well as the choice and number of unfair offers and time delays used (see Table S1for a discussion of this variability).

The previous studies using SHH with respect to cooperation have focused on short durations (seconds). For example, the studies by Rand et al [2], [6]–[7] examined changes in cooperation and retaliation (defection, free-riding) in seconds. In contrast, apart from one study [22] on acceptance rates in the UG, all the previous studies on UG acceptance rates have either had delays in minutes or hours (up to 24 hours) and none measured the time respondents took to respond in the immediate condition [18]–[24]. Furthermore, the one study with short response delays [22] did not delay responses longer than 10 seconds. While there is no specific theoretical significance of 10 seconds for systems 1 and 2 per se. Rand et al [2] identified 10 second as the median decision time in a PGG and that when manipulated to respond before or after 10 seconds the level of cooperation changed. As such, we incorporate delays of 1, 2, 8 and 15 seconds.

Furthermore, instructions to participants, in some of the previous UG studies, either explicitly changed the focus of the delayed condition to reconsidering of the original offers [19]–[20] or had an unrelated intervening task [17]. Asking for a reconsideration will, for example, increases the likelihood that people will change their answers [26].

Finally, it is not possible to examine the effect of the degree of unfairness, as many of these studies only looked at only one unfair offer structure (e.g., 80:20) [19] or had small Ns per offer [23].

Thus, while informative these studies do not allow us to examine specific predictions arising from SHH-FFSA hypothesis regarding responses to unfairness that are based on a shift from system 1 to system 2 over short time delays.

This study overcomes these design issues by using a fully factorial within subjects design manipulating (1) four enforced time delays prior to the accept or reject decision around the 10 second window (1, 2, 8 or 15 seconds) and (2) five offers (50:50, 60:40, 70:30, 80:20, 90:10) as well as (3) measuring the time taken to respond after the time constraint – the decision time, and (4) having no intervening tasks.

The main aim of this study is to examine the differential predictions from the combination of an SHH and a ‘fast to forgive-slow to anger’ (FFSA) heuristic (SHH-FFSA hypothesis), with the hypothesis arising from the ‘reflective model’: (1) economic temptation and (2) rationality. With respect to the SHH-FFSA hypothesis faster decisions should be associated with greater acceptance rates for mildly unfair offers (60:40) and lower acceptance rates for severely unfair offers (90:10). When responders are able to respond after short enforced delays they should accept more mildly unfair offers (‘fast to forgive’) than when given longer to decide (‘slow to anger/retaliate’). Thus, the SHH-FFSA suggests an interaction between degree of offer unfairness and time delays. The ‘reflective economic temptation hypothesis’ suggests that acceptance rates will be higher at short delays, reducing as the time delay increases, regardless of offer. In contrast the ‘reflective rationality hypothesis’ suggests that shorter delays should be associated with lower acceptance rates with acceptance increasing with longer time delays.

## Method

### Participants

204 undergraduate students (mean age = 20.4 years; SD = 3.8 years: 56% female) participated, all of whom played the role as the responder in a series of one-shot UG.

### The Ultimatum Game

**Figure 1:**
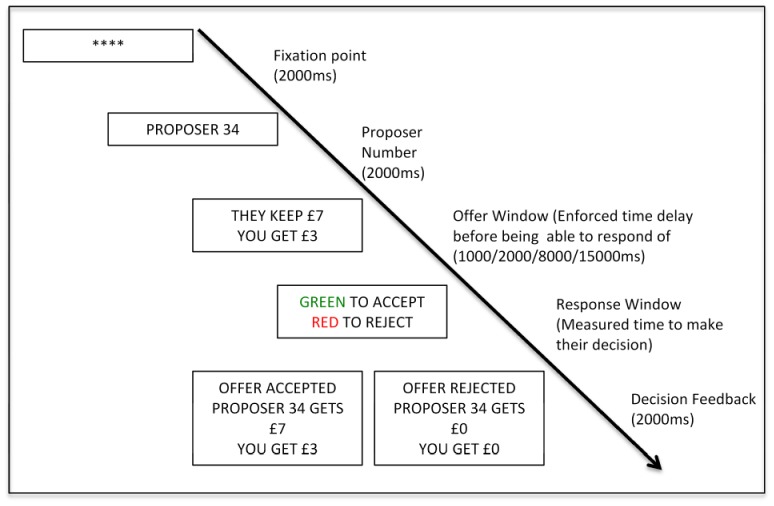
Schematic of the Ultimatum Game for the Responder.

A schematic representation of the responder's game is provided in Figure 1 (see Text S1 for instructions given to the participants). Initially on each trial an offer was presented with a fixation point for 2 seconds, followed by the number of the proposer who was making the offer (e.g., Proposer 34) for 2 seconds. Responders were then presented with the ‘offer window’ in the format “THEY KEEP £7, YOU GET £3”. The ‘offer window’ remained visible for the varying enforced time delays (1s, 2s, 8s, 15s) and during these time delays the participant was unable to make any response. After the time delay expired the ‘offer window’ was replaced with the ‘response window’. On the ‘response window’ the recipient indicated their decision to accept (by pressing a green key) or reject (by pressing a red key) the presented offer. The time taken to make the decision, while viewing the ‘response window’, was recorded as the post enforced time delay ‘decision time’. Thus, we experimentally controlled the time recipients had to wait before they could indicate their decision (the ‘time delay’) and measured the time it took after the time delay to indicate their decision (the ‘decision time’). A screen then reminded them of their decision – for example, if they accepted it said: ‘You Accepted. Proposer 34 gets £7 and you get £3’. This feedback was displayed for 2s. After this a new trial began.

The Ultimatum Game was programmed in E-Prime and consisted of 64 offers. 32 trials were of equal 50:50 splits. The other 32 were divided so that there were 8 trials each for a 60:40, 70:30, 80:20 and 90:10 splits. For the 50:50 offers there were 8 trials each with a 1, 2, 8, and 15 seconds enforced time delays, and for the 60:40, 70:30, 80:20 and 90:10 there were 2 trials at each enforced time delay. All participants completed the 64 trials, presented in a randomized order for each individual.

### Procedure

At the start of the experimental session participants were seated in an individual experimental cubical and were told that 64 students had been given £10 ($16.79) and asked how much they would give to another student. They were presented with written instructions explaining that they would be presented with each offer that these 64 students had suggested and had to decide whether to accept or reject it. Thus all participants played the role of the recipient. They were told that if they rejected the offer neither got the money and if they accepted it they and the proposer got the money in terms of the offer made. They were given an example trial. They were told that they were making real decisions and that after the experiment one of the trials would be selected at random – using a conditional information lottery design [27] – and they would be, and were, paid on the basis of that trial. All participants were paid. There was no show up fee. An experimenter was initially present to answer any questions. Once they understood the game, they completed all 64 trials in private. Proposers were identified by a number 1 through 64 (no photographs or names were used).

### Ethical Approval

The study received ethical approval from the participating Universities' School ethics boards (jm148-e35ec at Leicester University and AS/hcf 115 at Nottingham University). All participants were 17 years of age or over (range 17 to 55) and provided full written informed consent to take part in the study as approved by the ethics committees. Our ethical procedures conform to those of the British Psychological Society (http://www.bps.org.uk/sites/default/files/documents/code_of_human_research_ethics.pdf) and as such parental/guardian approval was not required for the one participant who was 17 years of age.

### Statistical Analysis

Given the within subject design, two-level random intercept regression models were specified in M*Plus* 7 [28], with enforced time delay (coded 1, 2, 8, and 15), degree of offer unfairness (coded 1, 2, 3, 4, 5 for 50:50 through 90:10 respectively) and decision time (group mean centered) as within subject predictors [29]. The outcome variable was the average proportion of offers accepted for each of the offers (50:50, 60:40, 70:30, 80:20, 90:10) for each of the 4 time delays (1 s, 2 s, 8 s & 15 s). Thus, there are 20 proportions per participant (5 offers by 4 time delays) resulting in a total of 4080 decisions across the 204 participants. Examining the distribution of these 4080 responses revealed that 98.8% of all the average proportions of offers accepted were either zero (32%), 0.50 (8.4%) or 1.0 (58.4%). Given the ordinal nature of these data we recoded the responses as 0 (zero and ≤0.49: 32.1%), 1 (≥0.50 and <1: 9.5%) and 2 ( = 1: 58.4%). These are converted back to proportions on a 0 to 1 scale for all graphs. Thus the two-level random intercept regressions were specified as ordered categorical regressions estimated using Maximum Likelihood with robust standard errors. Unless otherwise stated the reported regression coefficients are standardized coefficients.

All models were initially specified without post delay decision time. Post delay decision time was then added to examine if it added to the fit of the model both in terms of the overall model, as assessed by the Akiake Information Criteria (AIC: which should be smaller in the better fitting more parsimonious model) and in terms of its individual predictive power (i.e., was it an independent significant predictor?). If the addition of post delay decision time did not add to the overall fit or was non-significant the simpler model without post delay decision time was retained.

## Results

### UG Behavioural Data

**Figure 2:**
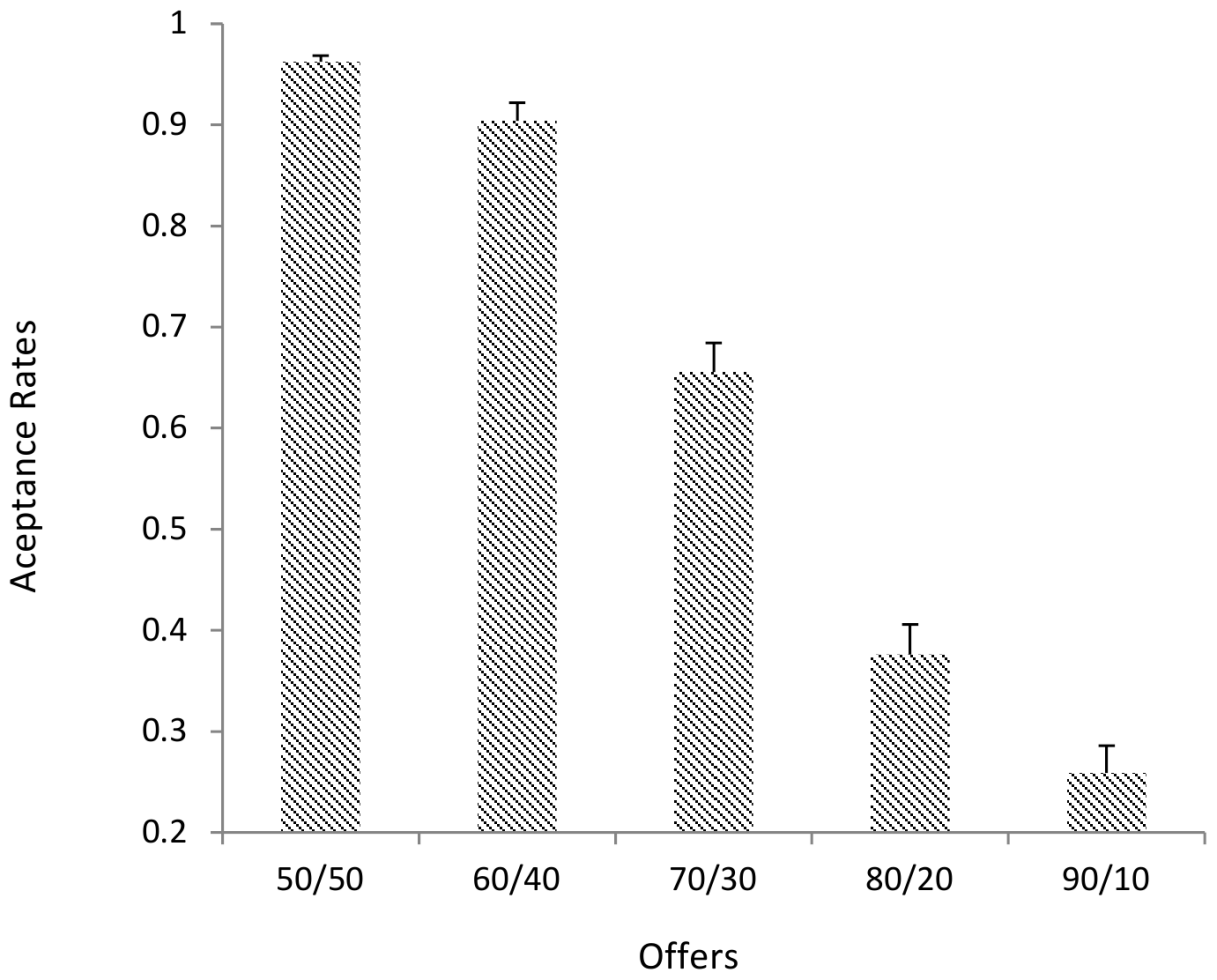
Average Acceptance Rates For Different Offers. Error bars  =  standard error of the mean.

Figure 2 shows that proportions of acceptance as a function of offers. A two-level random intercept regression of acceptance on offer showed a significant negative effect (β = −.893, *p*<.0001), indicating that as levels of unfairness increases levels of acceptance reduce.

### Associations with Decision Times

**Figure 3:**
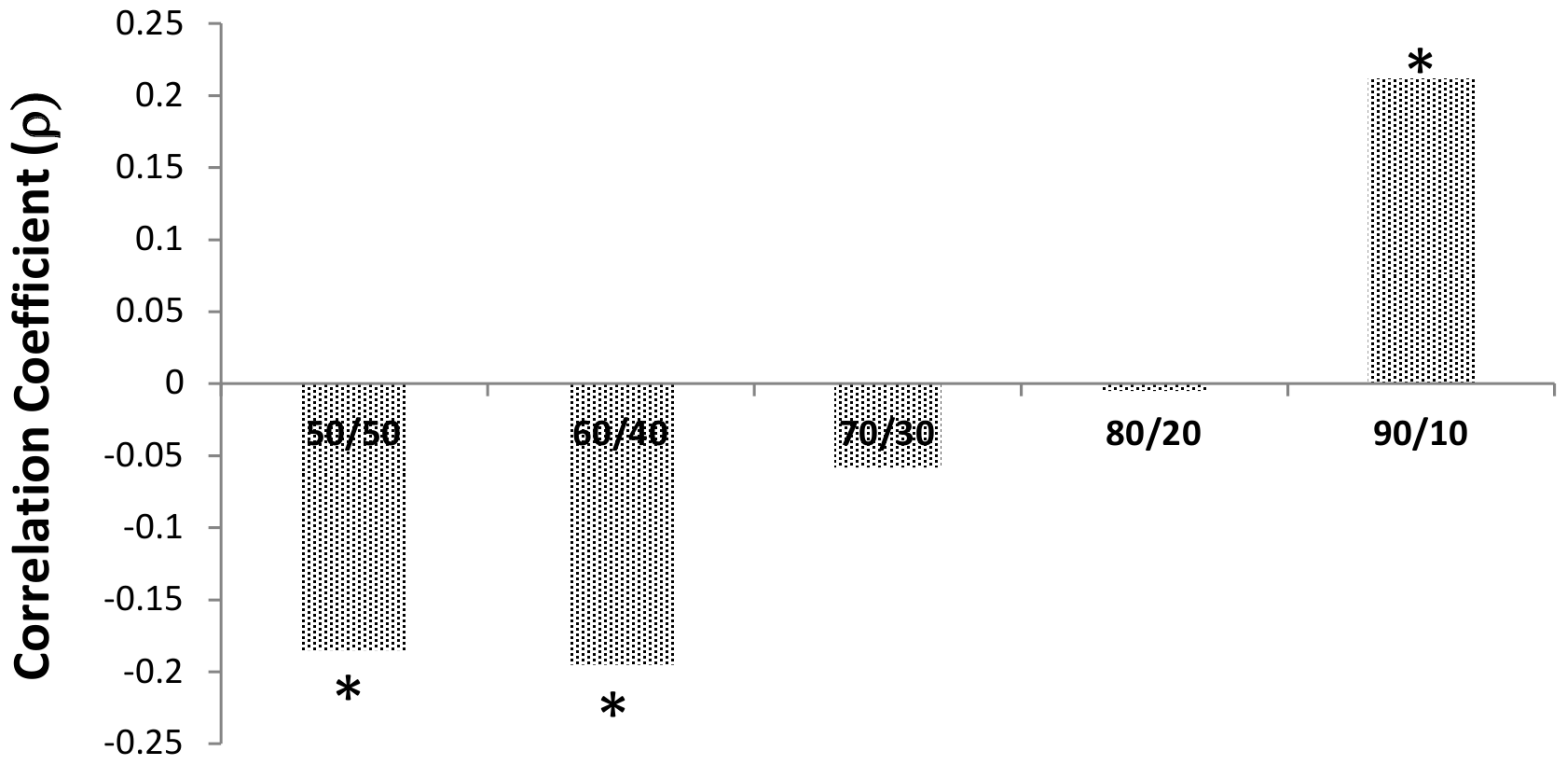
Correlations (Spearman's ρ) Between Post Delay Decision Times and Average Acceptance Rates Following the 1 Second Time Delays for the Different Offers. * *p*<.01

To examine the associations between post delay decision time and acceptance rates we isolated responses for the 1 second time delay as an index of the responders' general intuitive responding. For each offer we examined the correlation between post delay decision time (time taken to respond after the 1 second enforced time delay) and acceptance rates (see Figure 3). For the 50:50 offers the association was significant and negative (ρ = −.185, *p* = .008) as it was for the 60:40 offers (ρ = −.195, *p* = .005). Both indicate that faster responses were associated with greater acceptance. For the 70:30 (ρ = −.058, p = .406) and 80:20 offers (ρ = −.005 p = .942) the association was non-significant. However, for the 90:10 offers the association was positive and significant (ρ = .212, p = .002) indicating that faster responses were associated with lower acceptance or greater rejection. The significant associations for the 60:40 offers and the 90:10 offers were significantly different from each other (t _(201)_ = 4.02, p = .0001) as was the difference between the 50:50 offers and the 90:10 offers (t (201) = 3.06, *p* = .0025). Thus consistent with the prediction, the intuitive response for a mildly unfair offer (60:40) was to accept and for a severely unfair offer (90:10) was to reject. Also the results show clearly that the mildly unfair offers (60:40) are treated as though they are fair (50:50) offers. While consistent with the SHH-FFSA hypothesis these results are correlational and we cannot infer causality. Thus we also explored the effects of manipulating time delays experimentally.

### Manipulation of Time Delays

#### Time by All Offers

**Figure 4:**
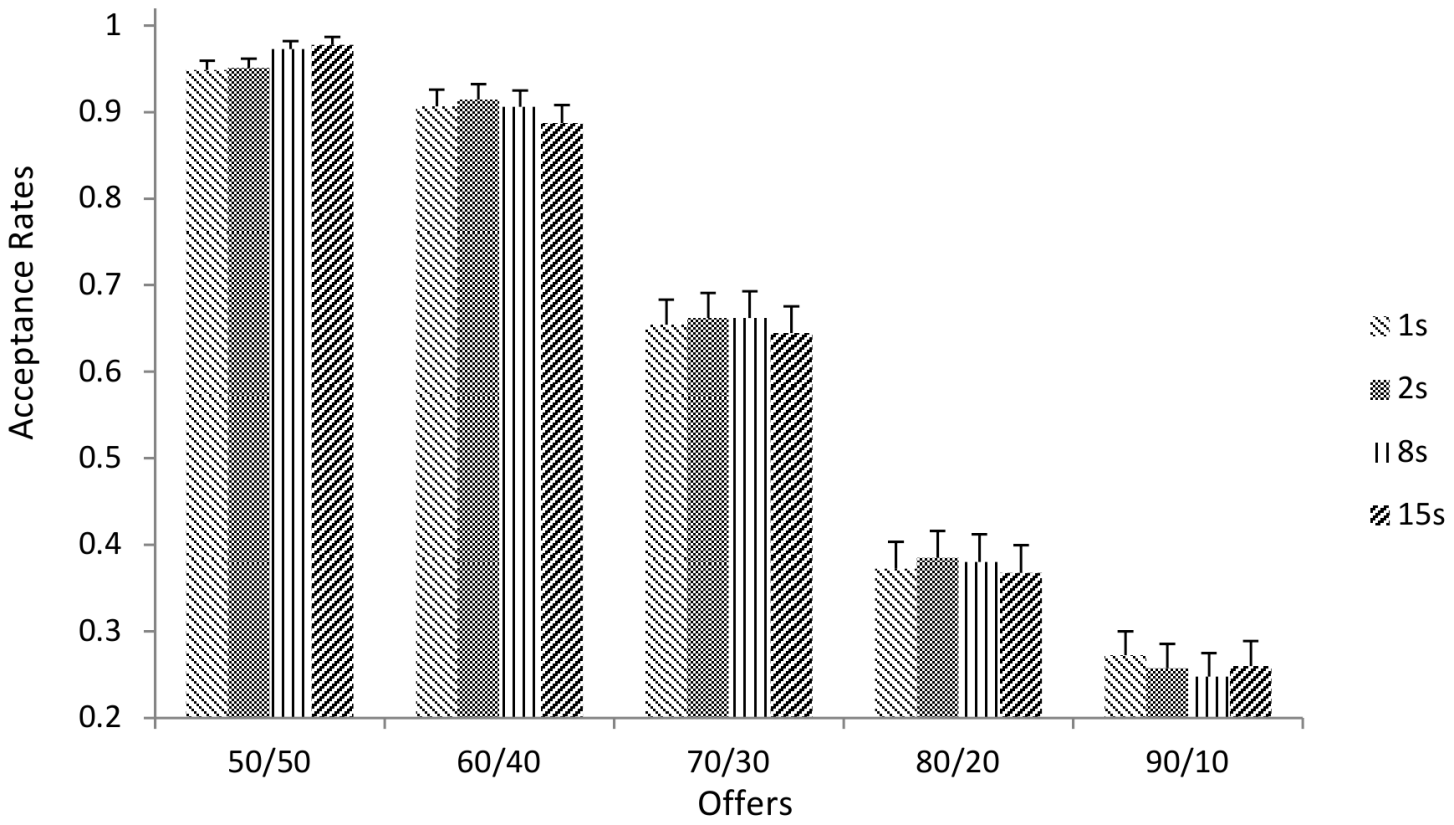
Average Acceptance rates for Different Time Delays for Each Offer. Error bars  =  standard error of the mean.

In the initial two-level random intercept regression model we regressed acceptance onto enforced time delays (1, 2, 8 and 15 secs), offers (50:50 to 90:10) and the interaction between the two (Figure 4). To aid the interpretation of the conditional effects in the presence of the interaction, time delays were recoded (0, 1, 7, 14) and offers recoded (0, 1, 2, 3, 4) [30]. This did not alter the overall pattern of results. The results showed a significant negative effect for offer (β = −.861, *p*<.0001), indicating that acceptance rates became lower as offers became more unfair, a non-significant effect for time delays (β = .06, p.094) and as predicted by the SHH-FFSA a significant interaction (β = −.071, *p* = .0285 [one tailed, as the interaction was predicted]). The model AIC was 3654.162. Adding post delay decision time (group mean centered) to this model resulted in a marginally worse fit (AIC = 3656.021) and the effect of decision time was non-significant (*p* = .78). As such, the initial model without post delay decision time was retained. A pattern of results for time delays (while approaching significance) is consistent with the ‘reflective rationality hypothesis’. However, the SHH-FFSA hypothesis suggests that at short time delays acceptance rates should be higher for mildly unfair offers (e.g., 60:40) reducing with longer time delays. Therefore, given the time by offer interaction we examined the effect of time for each of the offers separately.

#### 50:50 Offers

**Figure 5:**
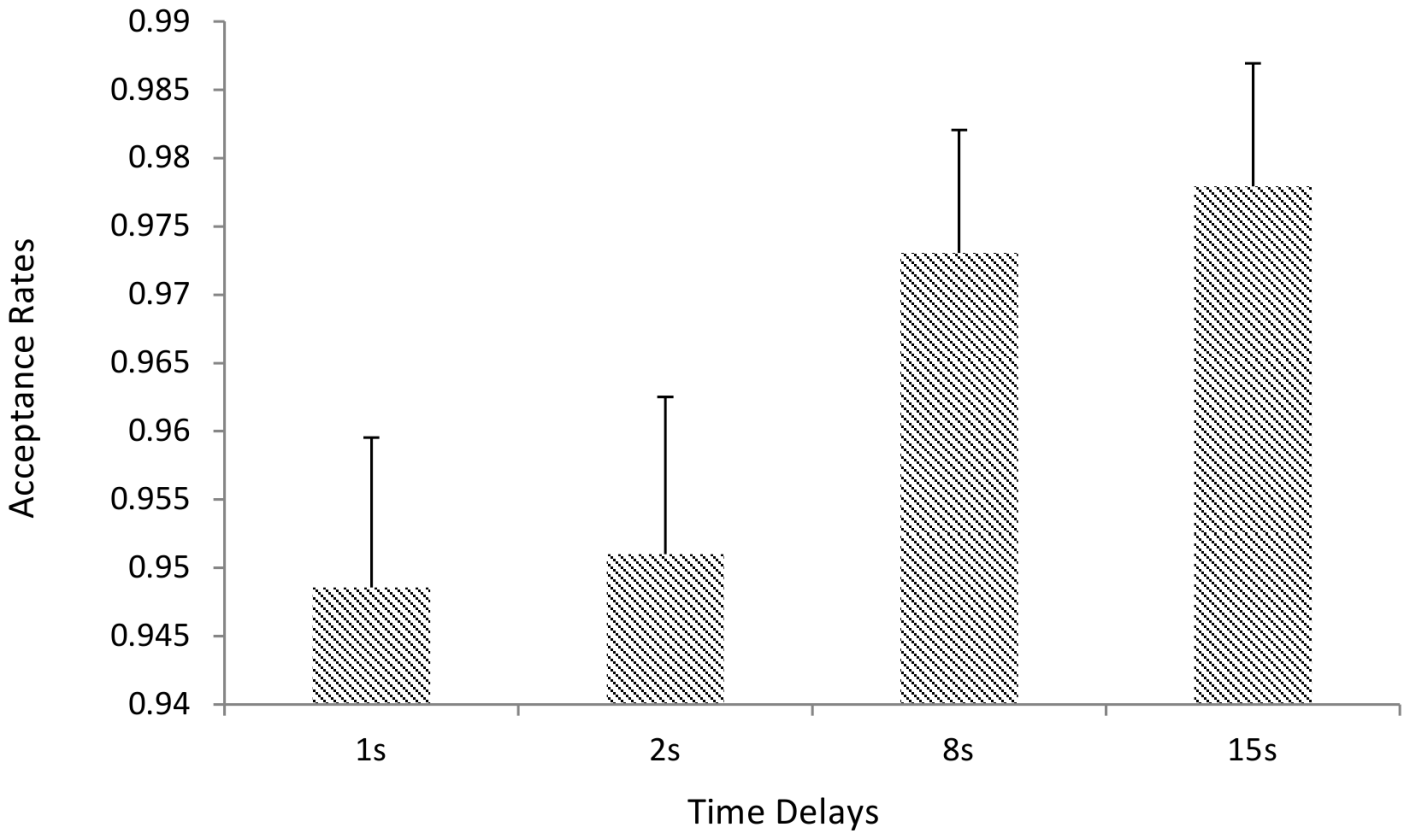
Average Acceptance rates for Different Time Delays for 50:50 Offers. Error bars  =  standard error of the mean.

We regressed acceptance onto the enforced time delays (coded: 1, 2, 8, 15) for the 50:50 offers only. There was a significant effect for time delays (β = .328, *p* = .002) indicating that for fair offers acceptance rates were greater with longer time delays (Figure 5). The model AIC was 383.469. Adding in post delay decision time did not improve the fit (AIC = 384.59) and the effect of post delay decision time was non-significant (*p* = .445).

#### 60:40 Offers

**Figure 6:**
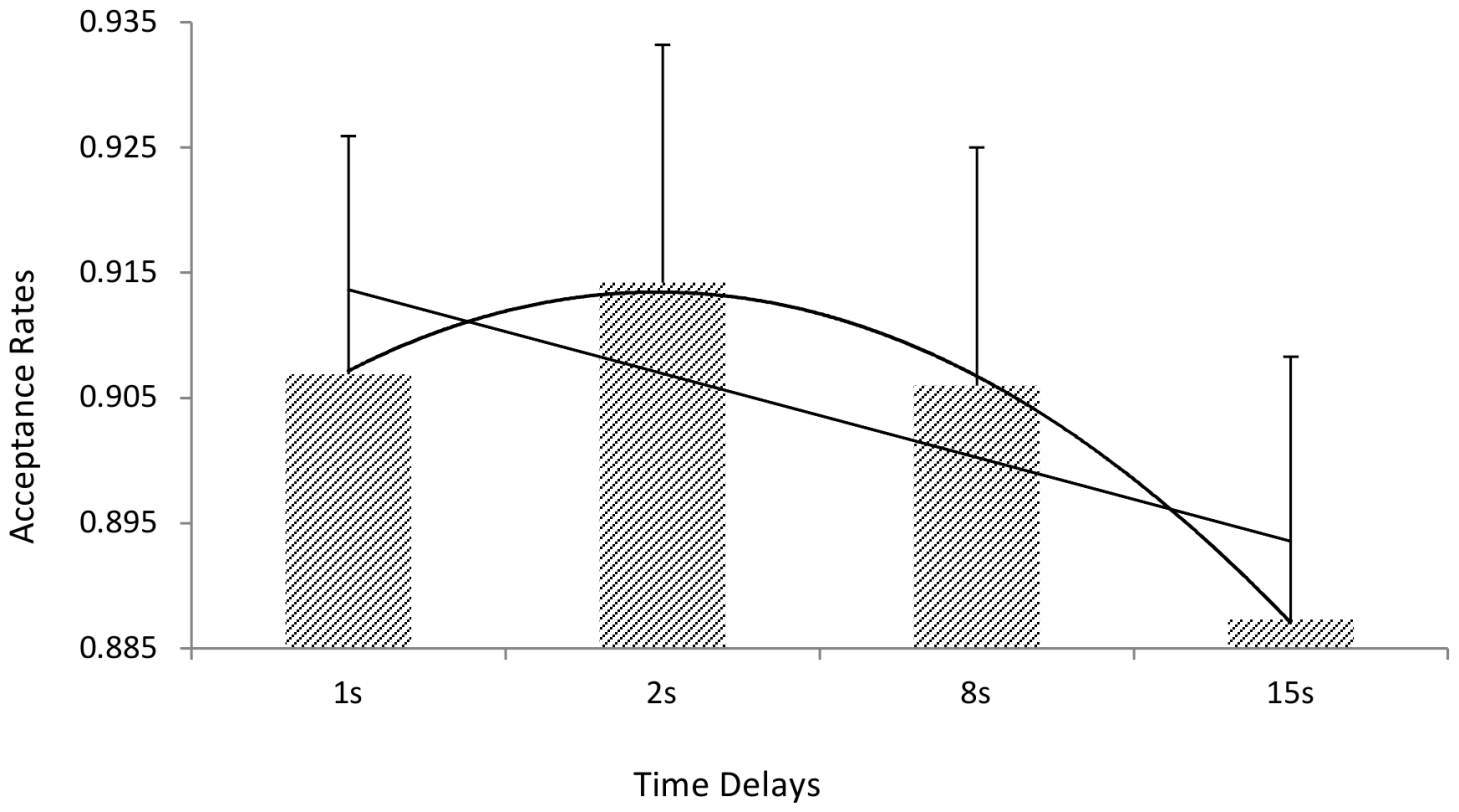
Average Acceptance rates for Different Time Delays for 60:40 Offers. Error bars  =  standard error of the mean. [Best fitting linear and quadratic trend lines included].

Regressing acceptance onto the enforced time delays (coded: 1, 2, 8, 15) resulted in a significant and negative effect (β = −.250, *p* = .033: AIC = 409.564) indicating that shorter time delays were associated with greater acceptance rates (Figure 6). Adding in post delay decision time did not improve the fit (AIC = 411.49) and the effect of post delay decision time was non-significant (*p* = .86). Examining Figure 6 suggests that the effect of time may be quadratic. Fitting a quadratic term indicated a significant effect ((β = −.262, *p* = .017: AIC = 408.796), which was a marginally better fit (AIC) than the linear model. Either way, longer time delays are associated with reduced acceptance rate for mildly unfair offers.

#### 70:30 Offers

There was no significant association between acceptance and enforced time delays (coded: 1, 2, 8, 15) (β = −.05, *p* = .454: AIC = 926.306). Adding in post delay decision time did not improve the fit (AIC = 927.71) and the effect of post delay decision time was non-significant (*p* = .42).

#### 80:20 Offers

There was no significant association between acceptance and enforced time delays (coded: 1, 2, 8, 15) (β = −.04, *p* = .570: AIC = 914.678). Adding in post delay decision time did not improve the fit (AIC = 916.220) and the effect of post delay decision time was non-significant (*p* = .53).

#### 90:10 Offers

There was no significant association between acceptance and enforced time delays (coded: 1, 2, 8, 15) (β = −.068, *p* = .329: AIC = 824.228). Adding in post delay decision time did not improve the fit (AIC = 916.220) and the effect of post delay decision time was non-significant (*p* = .212).

Thus consistent with the predictions, responders are more likely to accept with shorter delays, than longer delays, for mildly unfair offers (60:40) only. This suggests that a ‘fast to forgive, slow to anger’ heuristic may be operating [14]. However, we also observed that for fair (50:50) offers that acceptance increased with longer time delays. We did not predict this a-priori but offer an explanation in the discussion.

## Discussion

With respect to unfair offers (60:40 to 90:10) the pattern of results supports the combination of ‘SHH’ with a ‘fast to forgive, slow to anger’ heuristic for mild unfairness [6], [14]. For mild levels of unfairness the intuitive response is to accept (fast to forgive) and only with a longer time delays do rejection rates increase (slow to anger/retaliate). However, when unfairness is severe (90:10) the intuition is to reject. The ‘reflective economic temptation hypothesis’ offers a potential explanation of the pattern of results observed for the 60:40 offer. This hypothesis suggests that the higher initial acceptance rate reflects the selfish impulse to accept and it is only with the control exerted by system 2 that the cultural normative response – to reject unfairness – is observed [15]. We, however, feel that the ‘SHH-FFSA’ hypothesis offers a more parsimonious account. First, the SHH-FFSA hypothesis suggests that this pattern is only observed when the degree of unfairness is mild (uncertain), but when the degree of unfairness is severe (and certain) the intuitive response is always to reject. The ‘reflective economic temptation hypothesis’ does not make any particular prediction about the degree of unfairness. Second, the SHH-FFSA integrates two theoretical perspectives within a single framework. Third, this pattern of responding makes some evolutionary sense. When facing offers that are mildly unfair it is better, in terms of long term interactions, to repair quickly and get the interaction back to cooperation, a process that may also enhance reputation [10], [31]–[32]. However, when the situation is clearly unfair the optimal strategy is to reject, as this should lead to future transactions with higher and fairer offers [2]. However, it could be argued that intuitively cooperating, when offers are mildly unfair, may be disadvantageous leading to exploitation [33]. Indeed, some individuals may always cooperate, even when it is not advantageous to do so, and be exploited and indeed others may also be prone to exploit [11], [33]. However, on average, across encounters, the pattern identified here should be advantageous. On the other hand some individuals may always free-ride [34], and this highlights the importance of examining individual differences in strategy and personality with respect to positive and negative reciprocity [35].

With respect to fair offers (50:50) we observed a pattern of responses such that people are not only very likely to accept, with faster decision times linked to greater acceptance, but also that levels of acceptance increase with longer time delays prior to making the decision. Knoch et al [15] offer a suggestion that fits with this pattern. They suggest that for unfair offers in an UG, systems 1 and 2 are in conflict, but that this conflict is not present for fair offers in the context of an UG. System 1 and system 2 are working together here rather than in opposition [7]. In the context of people being treated fairly, it is intuitive and beneficial for potential future interactions to be fair back. In terms of our cultural norms (reflected in system 2) it is appropriate and rational to accept fair offers. Thus the fast and intuitive response to accept fairness should strengthen with more time to reflect on it.

Other studies on acceptance rates have reported increasing acceptance rates with longer time delays [20], [23], [24]. However, while these studies differ on a number of criteria (see Table S1) their delay conditions were much longer, ranging from minutes [24] to a day [20], than the delay used in this study. This longer delay may allow for greater reflection and evaluation than is possible in the shorter time delays of the study reported here. However, in most social encounters decisions about how to respond initially to unfairness will be made quickly and our findings speak to this. Longer delays to ‘cool off’ may reflect financial decisions that may be more associated with reflecting on longer term bargains and contracts before accepting them.

## Conclusions

The finding reported here support the SSH- FFSA hypothesis with respect to decisions about unfair offers. When offers are mildly unfair, people are fast to forgive (they not only accept more mildly unfair offers after shorter time delays, but also those who respond faster to mildly unfair offers accept more). With longer time delays, more mildly unfair offers are rejected (slow to retaliate/anger). We suggest this a more parsimonious account than a ‘reflective economic temptation’ model which suggest that acceptance rates will drop with longer time delays, but does not have anything to say about how this would vary as a function of degree of unfairness. When offers are severely unfair, people reject regardless of the amount of time they have to respond. The ‘reflective rationality’ model suggests that people should accept more with longer time to think, this is not observed for unfair offers.

## Supporting Information

Figure S1 **Perceived Fairness of Offers in the Ulimatum Game.**
(DOCX)

Table S1 **Experimental Details of Previous Studies Examining Time Constraints in UG Responder Acceptance Rates.**
(DOCX)

Text S1 **UG on screen instructions.**
(DOCX)
