# Supplementary material for: Fast to Forgive, Slow to Retaliate: Intuitive Responses in the Ultimatum Game Depend on the Degree of Unfairness
Source: PLoS One. 2014 May 12;9(5):e96344. doi: 10.1371/journal.pone.0096344 (PMC4018360; doi:10.1371/journal.pone.0096344)
Supplement: Figure S1 — Perceived Fairness of Offers in the Ulimatum Game. (DOCX) [file pone.0096344.s001.docx]

**Figure S1: Perceived Fairness of Offers in the Ulimatum Game**

We surveyed 205 additional undergradutes (mean age 19 [SD = 3.2], percentage female = 82%) and asked them, using a strategy method, to indicate the degree of fairness (1 = unfair to 5 very fair) associated with 50:50, 60:40, 70:30 , 80:20, 90:10 offers. The results are shown in Figure S1.

A oneway repeated measures ANOVA showed that there was a significant effect for offer (F _Greenhouse-Geisser_ (4, 604.5) = 870.56, p < .0001) with offers becoming perceived as increasingly unfair as they move from 50:50 to 90:10 (linear trend: F _(1, 203)_ = 1892.51, p < .001).

**Figure S1:** Perceived Fairness as a Function of Offer. Errors = standard errors
