# Supplementary material for: Fast to Forgive, Slow to Retaliate: Intuitive Responses in the Ultimatum Game Depend on the Degree of Unfairness
Source: PLoS One. 2014 May 12;9(5):e96344. doi: 10.1371/journal.pone.0096344 (PMC4018360; doi:10.1371/journal.pone.0096344)
Supplement: Table S1 — Experimental Details of Previous Studies Examining Time Constraints in UG Responder Acceptance Rates. (DOCX) [file pone.0096344.s002.docx]

**Table S1**: Experimental Details of Previous Studies Examining Time Constraints in UG Responder Acceptance Rates

| **Study** | **Decision Time Assessed** | **Design and Manipulation of time** | **Significance of Effect** |
| --- | --- | --- | --- |
| Bosman et al. (2001) | No | Lab Based Between Subjects Design  $2 show up fee, which is independent of earnings  **Immediate Treatment** (N = 16 P-R pairs): Responders immediately on receiving their offer make their decision. Rs have completed another decision task prior to the UG  **Delayed** ‘**Cooling off’ Treatment** (N = 22 P-R pairs): Receive the offer 1 hour before deciding, and do another decision making task in the intervening hour.  **Participants played just R roles** | **Non-significant** |
| Rubinstein (2007) | Yes | Did not manipulate time, but rather measured how long it took for people to make accept or reject decisions for a 90:10 hyper unfair offer (N = 2620; 63% accept). | **Non-significant** |
| Osumi & Ohira (2010) | No | Lab based experiment  **Daley: R has to wait** 0.5, 3 and 8 secs between offer and cue to respond. Strategy method to expose 20 participant to 30 offers of 50:50 (50% of trials) and, 70:30, 80:20, 90:10 (the remaining 50% of trials).  Participant split into high and low responder on psychopathy scores  **Participant always played the R role** | **Non-significant** |
| Sutter et al. (2003) | No | Played 9 rounds. One offer was real and 8 were fictitious, but participant did not know which was which and treated all rounds as real. Pain only for the real round. N = 32 responders  **Immediate**: Had to make their decision within10secs (20 secs on round 1)  **Delayed:** Had up to 100 secs to make their decision but this was not binding and could make it quicker.  **Participants played just R roles** | **Mixed**  Significantly accept more with the delay but only on round one. The was no significant effects round 2-9 |
| **Study** | **Decision Time Assessed** | **Design and Manipulation of time** | **Significance of Effect** |
| Oechssler et al. (2007) | No | Online experiment: Using Mini-UG with Ps making one of two possible offers (**5:5 or 8:2**). 5:5 offers rejected less than 2.5% of the time. 1/3^rd^ chose the 8:2 offer (this unfair offers are low in frequency)  **Immediate decision**  **Delayed**: **24 hours** to reconsider and ‘cool off’.  Two ways to pay (1) **cash** (1 ‘Lotto- Euro’ = 1 euro; N = 94)) or (2) **lottery** (given lottery tickets that could be played to win 500 euro; N = 112)  Lottery condition is taken from Oechssler et al. (2006).  **Participants played just R roles** | **Mixed**  **Lottery**: Significant effect with greater acceptances with longer delays  **Cash**: No significant effect. |
| Grimm & Mengel (2011) | No | Lab based experiment: Offers not manipulated  **Immediate** (N = 84 P-R pairs): Rs make an immediate decision and spend 10 mins completing questionnaires  **Delayed** (N = 132 P-R pairs): Rs receive the offer and spend 10 mins to complete questionnaire before deciding  C**ontrol** (n = 126 P-R pairs): (no delay, but asked if want to reconsider). (min = 8.4 and max = 12.07 minutes in the delay condition)  **Participants play the role of R only** | **Mixed**  Significant effects for very unfair offers (90:10 and 80:20) but not for mildly unfair offers. Such that greater time delays leads to greater acceptance when the effect is significant. The Ns of responders are low 10 and 8 respectively. |
| **Study** | **Decision Time Assessed** | **Design and Manipulation of time** | **Significance of Effect** |
| Cappelletti et al. (2008) | No | Lab based experiment: Offers not manipulated.  **Immediate**: 30 secs to decide  **Delayed**: 180 secs to decide  Cognitive load was also manipulated. In high cognitive load condition Rs had to remember 5 3-digit number while making their decisions. In the low cognitive load condition did not have the memory task. This task was incentivized with a high incentive (0.30 euro per digit remembered) and low incentive (0.003 euro per digit remembered). Endowment was also varied. Participant has a high (15 euro; N = 351) or low (7 euro: N = 373) endowment based on performance on an earlier task.  **Participants played both P and R roles,** using a strategy method | **Significant**  High and Low endowment. Both show delays lead to greater rejections |
| Neo et al (2013) | No  No | Expt 2: Lab based experiment: Asymmetrical UG (each chip worth $0.15 to Ps and $0.05 to Rs)  **Immediate** (N = 22 P-R pairs)  **Delay** (N = 21 P-R pairs): 15 mins  Expt 3: Online experiment (*m*Turk): Asymmetrical UG (each chip worth $0.03 to Ps and $0.01 to Rs)  **Immediate** (N = 88 P-R pairs)  **Delay** (N = 84 P-R pairs): 15 mins | **Significant**  Greater acceptance rates in the delayed condition  **Significant**  Greater acceptance rates in the delayed condition |

*Note*: P = Proposer, R = Responder; P-R Pairs = Proposer-Responder Pairs

Table S1 above provides the details of the previous studies examining times delays on the responders behavior in the Ultimatum Game (UG).

**Non-Significant Results**: The non-significant results tend to have smaller P-R pairs (Bosman et al., 2001) or small Ns (Osumi & Ohira, 2010) and have additional tasks (Bosman et al., 2001) or did not manipulate time delays and used only extremely unfair offers (Rubinstein, 2007).

**Mixed Results**: These tend to have much more complex designs, manipulating not just, time delay, but also the number of rounds (Sutter et al, 2003), payment structure (Oechssler et al., 2007) and also have intervening tasks (Grimm & Mengel, 2011).

**Significant Results**: These had slightly larger Ns, altered the incentive structures, as well as endowment.

**References**

Bosman R, Sonnemans J, Zoelenberg M. (2001). Emotions, rejection, and cooling off in the ultimatum game. Available: <http://dare.uva.nl/document/156>. Accessed August, 2013).

Cappelletti D, Guth, W, Pioner, M. (2008). Being of two minds: an ultimatum experiment investigating affective processes. Jena Economic Research papers number 2008-048 (ISSN 1864-7057) Available: [www.jenecon.de](http://www.jenecon.de). Accessed August 2013.

Grimm V, Mengel F. (2011). Let me sleep on it: delay reduces rejection rates in ultimatum games. Econ Letts 111: 113-115

Neo WS, Yu M, Weber RA, Gonzalez,C. (2013). The effects of time delays in reciprocity games. J Econ Psychol 34: 20-35.

Oechssler J, Roider A, Schmitz P. (2007). Cooling-off in Negotiations - Does it work? Avalable: <http://www.wiwi.uni-frankfurt.de/kolloquium/ss07/roider.pdf> . Accessed: August, 2013.

Osumi T, Ohira H. (2010). The positive side of psychopathy: emotional detachment in psychopathy and rational decision-making in the ultimatum game. Pers Ind Diffs 49: 451-456.

Rubinstein A (2007). Instinctive and cognitive reasoning: A study of response times. Econ J 117: 1243-1259.

Sutter M, Kocher M, Stranβ S. (2003). Bargaining under time pressure in an experimental ultimatum game. Econ Letts 81: 341-347.
