# Supplementary material for: Fast to Forgive, Slow to Retaliate: Intuitive Responses in the Ultimatum Game Depend on the Degree of Unfairness
Source: PLoS One. 2014 May 12;9(5):e96344. doi: 10.1371/journal.pone.0096344 (PMC4018360; doi:10.1371/journal.pone.0096344)
Supplement: Text S1 — UG on screen instructions. (DOCX) [file pone.0096344.s003.docx]

**Text S1: UG on screen instructions**

Instructions appearing on the screen for the ultimatum game prior to the start of the 64 games

Thank you for agreeing to take part in this study.

Prior to this study we surveyed 64 students who were given £10 and asked to say how much of the £10 they would be willing to give away to another person and how much they would keep for themselves. In the case of this study the other person is you

Please press 'SPACE' to continue

In this study you will be presented with each of the offers the 64 proposers made. You will be told how much each proposer decided to keep for themselves and how much to give away to you.

You have to decide if you accept or reject each offer. For example, the proposer may have decided to keep £7 and offer you £3.

If you accept you both get to keep the money (they keep £7 and you get £3).

If you reject the offer you both get nothing.

Please press 'SPACE' to continue

You will be presented with series of 64 offers for which you decide to accept or reject.

If you wish to accept an offer press the GREEN key

If you wish to reject an offer press RED key

One of your decisions will be selected at random at the end of the study and you will be paid based on that decision. Both you and the proposer will be paid the exact amount of money from that offer.

Please press 'SPACE' to continue

If you have any questions about the procedure please ask them now.

When asked to make your decision please respond as quickly as possible

When you are ready to begin please press 'SPACE'
